# Impact of the COVID‐19 pandemic on recruitment to clinical research studies in rheumatology

**DOI:** 10.1002/msc.1561

**Published:** 2021-05-03

**Authors:** Mazin Mirza, Stefan Siebert, Arthur Pratt, Elspeth Insch, Frances McIntosh, John Paton, Claire Wright, Christopher D. Buckley, John Isaacs, Iain B. McInnes, Karim Raza, Marie Falahee

**Affiliations:** ^1^ Sandwell and West Birmingham NHS Trust Birmingham UK; ^2^ Institute of Infection, Immunity and Inflammation University of Glasgow Glasgow UK; ^3^ Research into Inflammatory Arthritis Centre (RACE) Versus Arthritis UK; ^4^ Translational and Clinical Research Institute Newcastle University Newcastle upon Tyne UK; ^5^ Newcastle upon Tyne Hospitals NHS Foundation Trust Newcastle upon Tyne UK; ^6^ Rheumatology Research Group Institute of Inflammation and Ageing College of Medical and Dental Sciences University of Birmingham Birmingham UK; ^7^ Kennedy Institute of Rheumatology University of Oxford Oxford UK; ^8^ NIHR Birmingham Biomedical Research Centre University Hospitals Birmingham NHS Foundation Trust and University of Birmingham Birmingham UK; ^9^ Translational and Clinical Research Institute and Musculoskeletal Unit Newcastle University Newcastle upon Tyne UK; ^10^ MRC Versus Arthritis Centre for Musculoskeletal Ageing Research University of Birmingham Birmingham UK

**Keywords:** Clinical research, participant recruitment, rheumatoid arthritis, COVID‐19 pandemic

## 1 INTRODUCTION

Recruitment to clinical studies is often challenging, and there has been increasing focus on developing strategies to promote participant recruitment (Treweek et al., 2018). During the current pandemic, the recruitment of participants to non‐COVID‐19‐related clinical studies has been negatively impacted by issues including prioritisation of COVID‐19 research, redeployment of research staff and the need for social distancing (Mitchell et al., 2020). Almost 90% of the National Institute for Healthcare Research non‐commercial research was paused in 2020 (Iacobucci, 2020).

Anxieties relating to the pandemic have been elevated amongst the rheumatology patient community, particularly in relation to infection risk due to immunosuppressive treatment, self‐isolation, shielding and difficulty accessing usual care (Glintborg et al., 2021). Treatment decision‐making has also been negatively impacted, and healthcare professionals are less likely to initiate or step up treatment (Dejaco et al., 2021). These concerns are likely to exacerbate reluctance to participate in clinical research. It is likely that such behavioural and perceptual changes will continue to have an impact beyond the pandemic.

To assess the likely impact on recruitment to clinical research studies in rheumatoid arthritis (RA) and to identify strategies to overcome barriers to clinical recruitment, the management committee of the Research into Inflammatory Arthritis Centre Versus Arthritis (RACE: www.race‐gbn.org) conducted an online survey of patients with a diagnosis of RA which was distributed to the membership of the National Society for Rheumatoid Arthritis (NRAS), the UK patient‐led organisation specialising in RA and juvenile idiopathic arthritis (JIA).

## 2 METHOD

An anonymous online survey was conducted between 11 September 2020 and 6 November 2020. The survey questions were developed and agreed upon by consensus between researchers (including clinical researchers) and patient research partners affiliated with RACE. The survey platform used was Jisc Online Surveys®. A link to the survey was advertised via NRAS newsletters for members and social media platforms. Participants were included if they were aged 18 or older, and reported that they had a confirmed diagnosis of RA. The study included two vignettes, each describing a clinical rheumatological research study—an observational study and a trial of an investigational medical product—where participants were asked to indicate how likely they would be to take part in the study (a) before the COVID‐19 pandemic and (b) during COVID‐19 pandemic. Differences in the distribution scores before and during the pandemic were tested using the Wilcoxon signed‐ranks test. Participants were also asked to indicate the extent to which several factors, identified by the research team as having the potential to mitigate the pandemic's constraining effects, would have a positive or negative impact on their decision‐making about research participation. Finally, they were given the opportunity to provide open‐text responses, which were grouped into categories independently by MM and MF. Any discrepancies between themes were resolved via discussion between MM, MF and KR.

## 3 RESULTS

A total of 1002 respondents completed the survey. The majority were female (90.3%), of white ethnicity (96.2%), and aged 46–75 years (74.5%). Additional data on participant characteristics are summarized in Table S1. 43.3% reported they were currently taking biologic therapy, 76.8% reported taking one or more conventional disease‐modifying anti‐rheumatic drug (DMARD) and 17.5% reported current glucocorticoid therapy. Only 2.9% reported that they were not currently taking any medication for their RA. 81.4% of all participants had never participated in research studies like the hypothetical ones described in the survey.

Respondents were more likely to take part in an observational study than an interventional study both before (*z* = 15.89; *p* < 0.001) and during (*z* = 13.83; *p* < 0.001) the pandemic. Respondents were less inclined to take part in an observational research study during COVID‐19 compared to before the pandemic (*z* = 15.741; *p* < 0.001), with 86% of respondents stating that they were likely/very likely to take part in a study of this kind before the pandemic, and 64% indicating they were likely/very likely to take part during the pandemic (Table 1). Respondents were also less willing to take part in an interventional study during the pandemic than before (*z* = 14.715; *P* < 0.001), with 61% of respondents reporting that they were likely/very likely to take part before the pandemic, and 44% reporting they were likely/very likely to take part during the pandemic.

**TABLE 1:** Reported likelihood of taking part in (a) an observational study and (b) an interventional study before the pandemic and whilst COVID‐19 is present in the community

(a) Observational study scenario: “Imagine that you are considering taking part in a rheumatology research study that involves visiting a hospital every six months for two years. At each visit you may be asked to provide information about your health, and biological samples (e.g. blood, urine, joint tissue obtained by biopsy). The study does not involve taking an experimental drug treatment.”			
Likelihood of participation	Pre‐COVID‐19 pandemic	While COVID‐19 is present in the community	Percentage change during COVID‐19
Very likely	52.2%	30.9%	−21.3%
Likely	33.8%	33%	−0.8%
Neither likely nor unlikely	7.2%	9.6%	2.4%
Unlikely	4.5%	14.2%	9.7
Very unlikely	1.5%	10.9%	9.4
Don't know	0.8%	1.4%	0.6%

(b) Interventional study scenario: Imagine that you are considering taking part in a rheumatology research study that involves visiting a hospital every six months for two years. At each visit you may be asked to provide information about your health, and biological samples (e.g., blood, urine, joint tissue obtained by biopsy). This **involves an experimental treatment that may be more effective than the treatment you are currently taking for your RA**			
Likelihood of participation	Pre‐COVID 19	While COVID‐19 is present in the community	Percentage change during COVID‐19
Very likely	30.5%	19.6%	−10.9
Likely	30.4%	24%	−6.4
Neither likely nor unlikely	13.2%	13.7%	0.5%
Unlikely	16.3%	20.5%	4.2
Very unlikely	6.1%	19%	12.9
Don't know	3.5%	3.4%	−0.1%

Most participants responded positively or very positively to all suggested interventions to facilitate research participation whilst COVID‐19 was present in the community (Table 2), with over 75% responding that interventions such as remote study visits and information about safety precautions would have a positive or very positive impact on participation. Sixty‐two open‐text responses were recorded, with 13 of these relating to COVID‐19. Responses related to the pandemic included suggestions to minimize personal contact (e.g., clinic visits scheduled for quiet times of day or at non‐hospital sites), avoidance of public transport and the need for provision of detailed information about precautions to minimize risk of infection (including details of COVID‐19 testing arrangements for research staff and provision of a named contact for further support). Responses unrelated to the pandemic involved concerns regarding undergoing a synovial biopsy procedure, the safety of interventional medicinal products and their impact on existing treatment, and travel costs/difficulties. Positive comments focussed on the potential of the study to benefit patients' lives. Illustrative examples of open‐text responses are included in Table 2 and a full list of responses is available in Table S2.

**TABLE 2:** Factors that might influence participation in future rheumatology studies

How much would each of the following have a positive/negative impact on whether you would take part in rheumatology research studies like the ones described above whilst COVID‐19 is present in the community						
	Very positive	Positive	Neutral	Negative	Very negative	Don't know
Some research visits, but not all, can occur remotely via telephone	39.5%	41.3%	14.2%	4%	0.4%	0.6%
Some research visits, but not all, can occur remotely via video	34.9%	40.4%	17.6%	5.5%	0.7%	0.9%
Written information about COVID‐19 safety arrangements for hospital visits	36.8%	42.8%	16.1%	3.3%	0.3%	0.7%
Video about COVID‐19 safety arrangements for hospital visits	30.8%	37.7%	26.3%	4%	0.3%	0.8%
Written information about any biopsy/drug treatment that is required	41.8%	42.3%	11.2%	3.2%	0.7%	0.8%
Video about any biopsy/drug treatment that is required	36%	40.2%	18.3%	3.9%	0.8%	0.8%
Information about how the study results will be made available to participants	42.9%	44.1%	11.5%	0.8%	0.1%	0.6%
Information about how the study results will be made available to the public	36.2%	41.8%	19.7%	1.2%	0.4%	0.7%

Other		
	Category	Verbatim examples of open text responses
COVID‐19‐related (13 responses)	Need to minimize face to face contact	“Research visits to hospital at quiet times—e.g., evenings or weekends ‐ to minimise encounters with other people.”
	Need for information about safety precautions	“If I can be sure that the people I meet at the hospital are COVID‐19 tested, I would definitely take part.”
	Preference for remote/local study sites	“The only thing that would have a positive impact is if the study was entirely remote or if the medical site visited was my GP surgery (which I have far more confidence I can visit safely).”
	Need to avoid public transport	“COVID controls (knowledge of) is key, transport to hospital also key ‐ either provided with COVID controls, walkable or by car with free parking or would need to be very confident in public transport.”
Non‐COVID‐19‐related (49 responses)	Logistical concerns, e.g., travel, costs	“Distance from myself to hospital”
	Synovial biopsy concerns	“The biopsy part of this trial would make me unlikely to want to participate”
	Trial drug concerns	“For me, the potential risk of taking a trial drug would be a far more significant factor in my decision than the risk of catching COVID (which I trust would be minimised as far as possible by the research/medical staff).”
	Impact on current treatment	“Current status of my disease. If it is well controlled, I would be unlikely to want to risk disrupting that by introducing different treatments.”
	Desire to help	“Anything to help us find more support for this disease”

## 4 DISCUSSION

This study provides evidence that patients are less willing to consider participation in observational and interventional rheumatology research studies whilst COVID‐19 is present in the community. Most participants in our survey responded positively to all suggested factors and information that might reduce the perceived risks of research during the pandemic. By increasing opportunities for remote or non‐hospital‐based research visits, addressing concerns about minimising personal contact and avoidance of public transport, and providing clear information regarding safety precautions and how study results will be made available to the public, the impact of the pandemic on recruitment to clinical studies could be reduced. The open‐text responses provided several specific suggestions such as scheduling hospital visits at quiet times, use of mobile units outside of the hospital and regular COVID‐19 testing for research staff.

Participants were more inclined to participate in observational studies than they were for interventional studies, both before and during the pandemic. This may reflect non‐COVID‐related barriers to participation highlighted here, such as concerns about the safety of experimental medicinal products and their impact on existing treatment. Though most participants in this survey reported they would be willing to take part in clinical studies before the pandemic, only a minority of this sample had done so. Although we did not investigate the reasons for this, it may relate partly to a lack of opportunity (or awareness of opportunity) to participate.

Challenges in recruitment to clinical research studies are a well‐recognised problem and certainly precede the COVID‐19 pandemic. For example, a report from 2015 showed that 19% of registered trials were closed or terminated due to insufficient participants (Carlisle et al., 2015). A review of interventions to improve recruitment to randomised trials identified only three effective strategies with high‐certainty evidence to support their use: (1) open trials rather than blinded, placebo trials; (2) telephone reminders to people who do not respond to a postal invitation and (3) using a tailored, user‐tested approach to develop patient information leaflets. These strategies were associated with absolute improvements in recruitment rates of 10%, 6% and 1%, respectively (Treweek et al., 2018). Recent reviews suggest online recruitment methods and the use of social media may be more effective than traditional in‐clinic/offline recruitment methods (Brøgger‐Mikkelsen et al., 2020; Darmawan et al., 2020). Given that the pandemic will likely compound the difficulty of recruiting patients, both directly, over at least the short to medium term, and indirectly longer term due to ongoing patient concerns and adaptations to healthcare services, the need for such innovative strategies will be even greater.

Contemporary studies will need to adapt their procedures in order to protect patients and staff during the pandemic and address ongoing concerns in the future. Possible adaptations highlighted in the current study reflect those suggested in other disease contexts, which include improved infection control measures (and effective communication about those), remote visits and virtual screening clinics (Beane et al., 2020; Collier et al., ). Lack of access to, or cost of, transport has previously been noted as a barrier to recruitment, and the present findings suggest this is even more salient during the pandemic when public transport is actively avoided and discouraged.

The strengths of this study include the large sample size, anonymous survey questions to promote accurate disclosure, and multi‐stakeholder survey design with input from patients, researchers, and healthcare professionals. A limitation of the survey relates to the pragmatic recruitment method. Members of patient organizations such as NRAS are unlikely to be representative of all RA patients. White female respondents between the ages of 46‐75 years old were overrepresented in the current study. Whilst this may be representative of a population that is likely to respond to online surveys of this kind, alternative methodologies are likely to be useful to explore the views of other groups. In particular, attention should be paid to Black, Asian and Minority Ethnic (BAME) groups who are already less likely to engage in clinical research (Smart & Harrison, 2017) and are more likely to be adversely affected by COVID‐19 (Sze et al., 2020). A further limitation is that most of this sample had no previous experience of taking part in clinical studies. This study did not directly address barriers to research participation in general, and further investigation is needed.

Barriers to research participation identified during other pandemics include ethical, economical and logistical factors (Sigfrid et al., 2020). The current study quantifies the likely impact of the COVID‐19 pandemic on the recruitment of individuals with RA to clinical rheumatology studies and highlights strategies that patients believe may increase their likelihood to participate in future research. This is of increasing relevance to researchers in rheumatology, as the impact of this pandemic on clinical studies is likely to persist beyond the presence of the virus in the community, and future pandemics are likely.

## CONFLICTS OF INTEREST

The authors declare no conflicts of interest.

## ETHICS STATEMENT

This study complies with the Declaration of Helsinki, and was approved by the University of Birmingham Science, Technology, Engineering and Mathematics Ethical Review Committee (ERN_20‐1208). Informed consent was obtained from all participants.

## AUTHOR CONTRIBUTIONS

*Formal analysis (lead); writing—original draft preparation (lead)*: Mazin Mirza. Conceptualisation (equal); Methodology; Writing—review and editing: Stefan Siebert. *Conceptualisation (equal); methodology; writing—review and editing*: Arthur Pratt. *Conceptualisation (equal); methodology; writing—review and editing*: Elspeth Insch. *Conceptualisation (equal); methodology; writing—review and editing*: Elspeth Insch. *Conceptualisation (equal); methodology; writing—review and editing*: John Paton. *Project management; writing—review and editing*: Claire Wright. *Conceptualisation (equal); writing—review and editing*: Christopher D. Buckley. *conceptualisation (equal); writing—review and editing*: John Isaacs. *conceptualisation (equal); Writing—review and editing*: Iain B. McInnes. *Conceptualisation (equal); methodology; formal analysis (support); original draft preparation (support)*: Karim Raza. Conceptualisation (equal); *methodology (lead); investigation (lead); formal analysis (support); original draft preparation (support)*: Marie Falahee.

## Supporting information

Supplementary Material

## Data Availability

Data available on request.
