# Supplementary material for: Impact of the COVID‐19 pandemic on recruitment to clinical research studies in rheumatology
Source: Musculoskeletal Care. 2021 May 3;20(1):209–13. doi: 10.1002/msc.1561 (PMC8242596; doi:10.1002/msc.1561)
Supplement: Supplementary file 1 — Supplementary Material [file MSC-20-209-s001.docx]

**Supplementary Table S1: Participant Characteristics.**

| **Age group (years)** | **Number** | **Percentage (%)** |
| --- | --- | --- |
| 18-30 | 32 | 3.2 |
| 31-45 | 182 | 18.3 |
| 46-60 | 383 | 38.5 |
| 61-75 | 358 | 36 |
| Over 75 | 39 | 3.9 |
| **Gender** | **Number** | **Percentage (%)** |
| Female | 887 | 90.3 |
| Male | 94 | 9.6 |
| Other | 1 | 0.1 |
| Prefer not to say |  |  |
| **Ethnicity** | **Number** | **Percentage (%)** |
| White | 952 | 96.2 |
| Black | 8 | 0.8 |
| Asian | 14 | 1.4 |
| Mixed | 5 | 0.5 |
| Other | 3 | 0.3 |
| Prefer not to say | 8 | 0.8 |
| **Response** | **Number** | **Percentage (%)** |
| I am not currently taking any medication for my rheumatoid arthritis | 29 | 2.9 |
| Steroids | 175 | 17.5 |
| Methotrexate | 595 | 59.4 |
| Hydroxychloroquine | 293 | 29.2 |
| Sulfasalazine | 210 | 21 |
| Biological therapy | 434 | 43.3 |
| Don’t know | 3 | 0.3 |
| Other | 138 | 13.8 |

**Supplementary Table S2:** Open text responses to factors that might influence participation in future clinical rheumatology studies.

| Number | Response |
| --- | --- |
| 1 | I believe hospitals are one of the safest places in our communities at the moment. |
| 2 | Research visits to hospital at quiet times - e.g. evenings or weekends - to minimise encounters with other people. |
| 3 | Full COVID-safe arrangements at the research venue. |
| 4 | My concerns are around going to a hospital during the pandemic. All information available is also key to my participation. |
| 5 | I would be reluctant to take part in any trials involving a new drug during the Covid pandemic because I’d be concerned about further compromising my immune system. I would be far more likely to cooperate with anything requiring a hospital visit if it could be held in a mobile unit vehicle such as those that women attend for mammogram appointments. In other words, I still wish to help medical research but I’m now more cautious. |
| 6 | Information about how the hospital is isolating Covid patients away from other patients ie how they are protecting our health |
| 7 | Having been to hospital for treatments during Covid, I’m not worried about going there. |
| 8 | Covid controls (knowledge of) is key, transport to hospital also key - either provided with Covid controls, walkable or by car with free parking or would need to be very confident in public transport. |
| 9 | If I can be sure that the people I meet at the hospital are Covid-19 tested, I would definitely take part. |
| 10 | Safety issues of attending hospital during covid |
| 11 | The only thing that would have a positive impact is if the study was entirely remote or if the medical site visited was my GP surgery (which I have far more confidence I can visit safely) |
| 12 | My decision on hospital visits would depend on covid infection in the area and hospital infection at the time. |
| 13 | Too disabled to manage visits to hospitals |
| 14 | I would need to know how far I would need to travel and whether expenses are paid if I had to travel away from local area |
| 15 | Where testing to take place |
| 16 | Would obviously be dependent on whether I was in a lockdown area |
| 17 | Going to a different setting that isn't a hospital |
| 18 | Distance from myself to hospital |
| 19 | Would not consider research where untried drugs are concerned |
| 20 | I would concider drug trials if there were assurences about its safety |
| 21 | Would have reservations about any research that involves drug treatments unless recommended by my rheumatology team |
| 22 | Current status of my disease. If it is well controlled, I would be unlikely to want to risk disrupting that by introducing different treatments |
| 23 | Part of my judgement is affected by the fact that my current treatment regime is working well rather than the Covid19 dimension |
| 24 | As long as i am safe and given relevant information i would behappy |
| 25 | Presumably my consultant would be involved in the decision about trying new drugs? |
| 26 | Information about the likely side effects of any drug treatment, any interactions with other medicines, how success will be measured, impact on other co-existing conditions, what to expect as a result of the trial and treatments and what proportion will be given a drug and what proportion a placebo. |
| 27 | For me, the potential risk of taking a trial drug would be a far more significant factor in my decision than the risk of catching covid (which I trust would be minimised as far as possible by the research/medical staff). |
| 28 | I would want information about the safety checks that had been carried out on any new drugs I might be asked to take. |
| 29 | If I were to take part in a study involving trialling a new medication, the most important thing to me would be to know how it would affect my day to day life, rather than COVID precautions. |
| 30 | I am happy with the result of asking Methotrexate and wouldn't want to take anything else which might have side effects unless I had to. |
| 31 | I would like full information on the possible drug. |
| 32 | Hard to say what I would do. Need to know whose going to take care of me should i get sick. Whose going to pay for my health care cost and other expenses |
| 33 | Not interested in being used for drug experiments |
| 34 | was prescribed drugs for RA, made me very ill |
| 35 | I would want to know all possible side effects of any trial drug before thinking of taking it, regardless of covid. The only other thing that would make me more likely to do a study is not having a covid risk (ie not going anywhere or having any medical staff visit my home) |
| 36 | Having taken so many RA drugs with little or no control achieved over the last 20 years, I would not take part in any drug trial. I now take xeljanz 5 mg BD and that is working well so far. |
| 37 | The more information the better |
| 38 | Anything to help us find more support for this disease |
| 39 | Anything that can help and stop someone from suffering this horrible disease that causes so many other problems in the future has to be a good think. |
| 40 | I would do anything that would help with this awful disease. |
| 41 | Needs more research to help everyone RA |
| 42 | I live in Jersey where the rheumatoid support is very low. I’d love to be part of a national survey which would tell me. Ore about my condition. |
| 43 | If it helps RA research for others, then fine. And does not put me at risk . |
| 44 | I would be willing to cooperate as I was only diagnosed 5 months ago (I had an idea was suffering same ailments as my mother suffers quite a lot with RA for about 20+ years. |
| 45 | being diagnosed at age 18 and now 49 it would be wonderful to have a cure. |
| 46 | I’m more than happy to take part. It would be my way of helping. |
| 47 | I am reluctant to have biopsies since infection and healing generally are a problem on the drugs I take |
| 48 | Not keen on skin biopsy as have had bone marrow tests for cancer, embarrassed to say wouldn't want to do drug trials |
| 49 | Biopsy would be a big hurdle... |
| 50 | The biopsy part of this trial would make me unlikely to want to participate |
| 51 | Not keen on biopsy if not medically necessary. But would be happy for scans,tests etc |
| 52 | It's the biopsy that puts me off and makes me feel nervous |
| 53 | Unable to think of anything else. |
| 54 | I can't say I have any problems about being involved in this study. |
| 55 | None |
| 56 | I get easily distracted watching videos and prefer written information. |
| 57 | I would prefer the belt and braces approach to cross reference and understand the information. A named contact and focus /reference group. |
| 58 | I have been put on a new biological drug which I inject myself every 2 weeks also inject Methotrexate every week |
| 59 | I hate watching videos |
| 60 | None |
| 61 | Unsure |
| 62 | Prefer one to one with doctors. Please bring back soon. I visit hairdresser! |
